# The NO_x_ Degradation Performance of Nano-TiO_2_ Coating for Asphalt Pavement

**DOI:** 10.3390/nano10050897

**Published:** 2020-05-08

**Authors:** Huanan Yu, Wan Dai, Guoping Qian, Xiangbing Gong, Dayao Zhou, Xi Li, Xinglin Zhou

**Affiliations:** 1 National Engineering Laboratory for Highway Maintenance Technology, Changsha University of Science & Technology, Changsha 410114, China; Huanan.yu@csust.edu.cn (H.Y.); xbgong@csust.edu.cn (X.G.); lixi@csust.edu.cn (X.L.); 2 School of Traffic and Transportation Engineering, Changsha University of Science & Technology, Changsha 410114, China; daiwan@stu.csust.edu.cn; 3 Hunan International Scientific and Technological Innovation Cooperation Base of Advanced Construction and Maintenance Technology of Highway, Changsha University of Science & Technology, Changsha 410114, China; 4 Guangdong Communication Planning & Design Institute Co., Ltd, Guangzhou 510507, China; zhoudayao0919@sina.com; 5 Automobile and Traffic Engineering, Wuhan University of Science and Technology, Wuhan 430081, China; zhouxinglin@wust.edu.cn

**Keywords:** pavement coating, photocatalytic material, NO_x_ degradation, silane coupling agent

## Abstract

The NO_x_ degradation performance of nano-TiO_2_ as a coating material for the road environment was evaluated in this research. The nano-TiO_2_ coating materials for both road surface and roadside were prepared by using anatase nano-TiO_2_, activated carbon powder, silane coupling agent and deionized water. The impact of varying amounts of coating material and silane coupling agent were evaluated. The road environment of NO_x_ degradation was simulated by the photocatalytic test system designed by the research team. For the road surface coating, the photocatalytic degradation experiments of NO under different radiation intensities were carried out. The results show that the material has good photocatalytic degradation performance, and the proper amount of silane coupling agent can enhance the bonding performance of the material and asphalt mixture. For the roadside coating, sodium dodecylbenzene sulfonate was selected as the surfactant to carry out the photocatalytic degradation experiment of NO_2_ with different dosages of surfactant. The results showed that when the mass ratio of nano-TiO_2_ and surfactant was about 1:2, the catalytic degradation effect of the material was the best.

## 1. Introduction

### 1.1. Background

Automobile exhaust is one of the most serious sources of air pollution, which can cause serious harm to both humans and the ecological environment, and which has become more and more serious with the increasing number of motor vehicles [1,2,3]. The elimination of environmental pollution caused by automobile exhaust has become a very important topic. Nano titanium dioxide (nano-TiO_2_) is a widely used nano material which is able to degrade pollutants because of its good photocatalytic oxidation function [4].

Since Fujishima and Honda used TiO_2_ as a catalyst to electrolyze water in 1972 [5], the research on nano-TiO_2_ photocatalyst has been increasing gradually. In 1984, Brown and Darwin evaluated the effect of methyl orange additive on the photocatalytic activity of TiO_2_ [6]. Cui et al. studied the influence of Nb_2_O_5_ on the activity of TiO_2_ [7]. Vorontsov et al. found that when TiO_2_ was used to oxidize carbon monoxide (CO) and nitrogen oxides (NO_x_), the reactive formation was nitrates and carbon dioxide (CO_2_), and it also found that TiO_2_ can recover its photocatalytic activity after surface cleaning [8].

Around the end of the 20th century, many places around the world, including Chiba of Japan in 1999 [9] and Milan of Italy in 2002 [10], have applied the nano-TiO_2_ in the construction of cement concrete pavement. The Antwerp City Park in Belgium constructed a sidewalk of about 10,000 m^2^ with cement concrete bricks containing nano-TiO_2_ [11]. In the protection of buildings and cultural heritage, TiO_2_ was often used as a stone coating for self-cleaning and biocidal purposes [12,13]. Liang et al. discussed the removal effect of nano-TiO_2_ mixed into porous concrete on rainwater runoff pollution [14]. Sikkema et al. analyzed the photocatalytic degradation efficiency of NO_2_ in different environments of cement concrete pavement containing TiO_2_ [15]. Mendoza et al. compared the mechanical properties and photocatalytic properties of TiO_2_ and TiO_2_-SiO_2_ coating cement [16]. He et al. compared the effect of two kinds of doping methods on the photocatalytic degradation performance of TiO_2_ particles [17].

Besides the above research and the of nano-TiO_2_ photocatalytic on cement concrete pavement, there is a lot of research on nano-TiO_2_ in the degradation of air pollution in asphalt pavement too, as more than 90% of the world’s new highway construction is asphalt pavement [18,19]. Tan et al. applied nano-TiO_2_ to asphalt pavement materials in the forms of coating and mixing [20]. Segundo et al. used the spray deposition method to prepare photocatalytic road sections [21]. It was found that nano-TiO_2_ particles and ZnO particles can make asphalt softer and have a better short-term aging resistance effect. Li et al. added nano materials to asphalt mixture and found that nano materials can enhance the viscoelasticity, high temperature stability, aging resistance and fatigue resistance of asphalt mixture [22]. Wang et al. evaluated the properties and preparation technology of the photocatalytic coating for asphalt pavement [23]. Based on the properties of the photocatalytic coating, the optimum content of TiO_2_ in the photocatalytic coating and the best spray content of the photocatalytic coating on the flat surface were obtained. Qian et al. evaluated the effect of SBS modified asphalt with different content of nano-TiO_2_ on the degradation of nitrogen and oxygen and also evaluated the effect of nano-TiO_2_ on the rheological properties of asphalt pavement [24]. Wang, et al. developed a novel method of TiO_2_ coating on asphalt pavement and a construction method of TiO_2_ modified aggregate coating photocatalytic air purification asphalt pavement [25,26]. The test results have shown that these methods can achieve a sustained NO_x_ degradation rate. da Rocha Segundo et al. used bulk incorporation and spray methods to produce asphalt mixture with photocatalytic function, and evaluated the influence of traffic and weathering wear on photocatalytic efficiency and mechanical properties [27]. Chen et al. studied the influence of N-doped TiO_2_ as a photocatalyst coating on asphalt pavement on the degradation of automobile exhaust and evaluated the photocatalysis and durability of N-doped TiO_2_ photocatalytic asphalt pavement materials [28].

In 2008, French researchers sprayed nano-TiO_2_ on the wall surface of three buildings and found that the coating wall can greatly reduce the concentration of NO_x_ compared with ordinary walls [29]. Babaizadeh and Hassan used life cycle assessment (LCA) to evaluate the application of the nano-TiO_2_ coating on residential glass and found that it had a certain effect on air purification [30]. Mendoza et al. analyzed and compared the efficiency of photocatalytic nano-TiO_2_ and WO_3_/TiO_2_ coating on the urban green infrastructure with different structural configurations (natural zeolite and permeable concrete block) for NO_x_ purification [31]. Sun et al. evaluated the effect of different adding methods and the same dosage of nano-TiO_2_ on the catalysis of harmful gases [32]. Shen et al. used a new method to prepare a kind of super smooth surface photocatalytic concrete [33], and found that the pollutants on its surface can be washed away by rainwater. This new type of concrete shows great potential in the self-cleaning facing materials of urban buildings.

It can be found from the literature review that most of the current research was mainly focused on the influence of the incorporation way, the amount of nano-TiO_2_, the temperature, and humidity on the catalytic degradation performance. However, studies on the impact of different amounts of surfactant and different levels of UV radiation intensity on the photocatalytic effect are few. Moreover, the NO_x_ concentration studied is quite different from than that in the actual road environment for many indoor experiments.

### 1.2. Objective

Based on the field monitoring of NO_x_ concentration in different periods of road environment in typical sections of Guangzhou (China), the photocatalytic degradation tests were conducted under different levels of UV radiation intensity using the self-developed photocatalytic environmental chamber, the degradation performance of the designed road surface coating and the roadside coating was tested. Additionally, the impact of different surfactant amounts was evaluated. 

The purpose was to evaluate the road surface and roadside coating styles, for the following reasons: because automobile exhaust is discharged closely to the road surface, and the NO_x_ concentration of the road surface is high, which could make the TiO_2_ degrade more NO_x_, so the road surface coating styles were evaluated. However, the road surface coating could not be sustained because it may rubbed off because of tire rotation, also the coating could impact the skid resistance, so the roadside coating style was evaluated.

**Figure 1:**
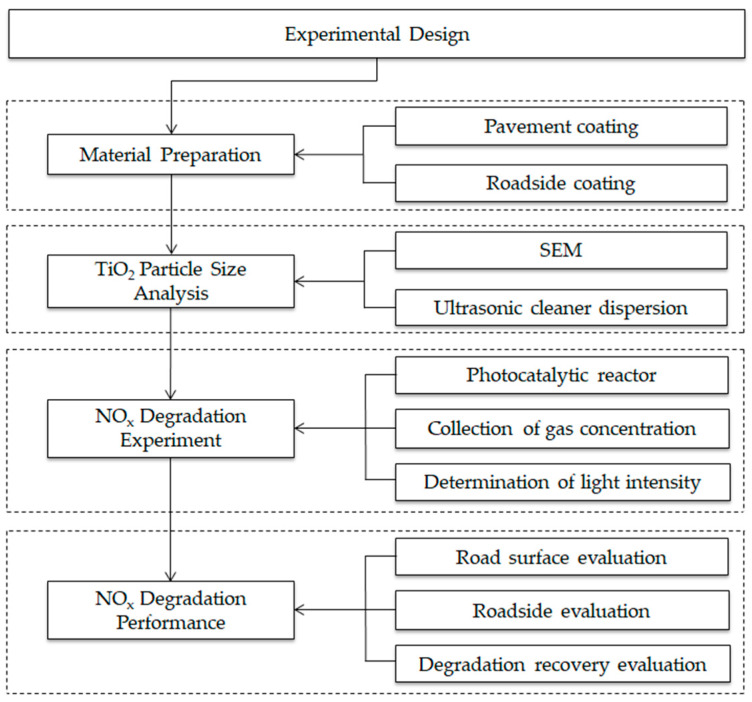
Research and experiment flowchart.

The experiment design and research flowchart are shown in Figure 1.

## 2. Materials and methods

### 2.1. Properties of Nano-TiO_2_

In this research, the VK-TA18 nano-TiO_2_ (Xuancheng JingRui new materials company, Anhui, China) was used, and the technical indicators were shown in Table 1.

The density was measured according to the Test Methods of Aggregate for Highway Engineering (China) and the measured result was about 3.132 g/cm^3^ [34]. The size of the nano-TiO_2_ was measured at room temperature of 13 °C. The procedures of measuring the nano-TiO_2_ sizes were as below: firstly, the nanoparticles were dispersed in anhydrous ethanol, and then put in the ultrasonic cleaner for dispersion for one hour (KQ5200DE numerical control ultrasonic cleaner produced by Kunshan Ultrasonic Instrument Company). The particle size analysis results shown that the minimum particle diameter is 71.0 nm, the mean particle diameter is 152.3 nm, and the median particle diameter is 139.9 nm. A scanning electron microscope (SEM) was also used to observe the nano particles and it found that only the size larger than 5 μm clumps can be observed, which indicated that the nano particles were agglomerated together during the transport and storage process, and which could affect the photocatalytic efficiency [24].

### 2.2. Material Preparation

#### 2.2.1. Raw Material

In order to improve the degradation efficiency of photocatalytic reaction, the active carbon powder of finer than 200 mesh was selected as the absorbent. With its huge specific surface area, the active carbon powder can greatly increase the contact area between photocatalyst and exhaust gas [35].

There are two different coating applications that were evaluated in this research, which including the road surface coating and the roadside coating. In order to prevent the coating material peeled off and increase the durability of the coating, the KH-550 silane coupling agent produced by Nanjing Pinning was used as the surface modifier which could enhance the adhesion between nano-TiO_2_ powder and asphalt mixture for road surface coating. Sodium dodecylbenzene sulfonate was used as the surface modifier for the roadside coating, and the deionized water was selected as the solvent.

#### 2.2.2. Road Coating Material Preparation

The road surface coating material was prepared below: firstly, 0.2 g of anatase nano-TiO_2_ and 0.2 g of activated carbon powder were mixed together by mechanical agitation, and 0.1 g of dispersant was added into 10 g deionized water to disperse the mixed powder. In order to evaluate the influence of different surface modifiers on the photocatalytic properties of TiO_2_, five different contents (0, 0.2, 0.4, 0.6 and 1.0 g) of KH-550 silane coupling agents were added into the solution, and then the five different road surface coating materials were dispersed with ultrasonic cleaner for 2 h. And then the coating material was spray coated at an amount of 250 g/m^2^ on the surface of the asphalt pavement specimens.

The roadside coating material was prepared as follows: firstly, 0.2 g of anatase nano-TiO_2_ and 0.1 g of dispersant were added into 10 g of absolute ethanol for disperse, and three different amount (0.2, 0.4 and 0.6 g) of sodium dodecylbenzene sulfonate were added respectively, and then three kinds of photocatalytic samples of roadside coating materials were prepared with ultrasonic cleaner by dispersing for 2 h. After the roadside coating material was prepared, the roadside coating material was coated on the surface of the sandwich plywood at dosage of 250 g/m^2^ through a spray method.

The photocatalytic degradation reaction of TiO_2_ can be expressed in the Equations (1) to (11):

(1) $${\mathrm{TiO}}_{2}\overset{\;\mathrm{hv}\;}{\to }e^{-}+h^{+}$$

(2) $$h^{+}+H_{2}O\;\overset{\;}{\to }\;{\text{·}\mathrm{OH}+H}^{+}$$

(3) $$e^{-}+O_{2}\;\to \;O_{2}^{-}\overset{{\;H}^{+}}{\to }{\mathrm{HO}}_{2}$$

(4) $${2\mathrm{HO}}_{2}\text{·}\to {\;O}_{2}+H_{2}O_{2}$$

(5) $$H_{2}O_{2}+\;O_{2}^{-}\;\to \text{·}\mathrm{OH}+OH^{-}+O_{2}$$

(6) $$H_{2}O_{2}\overset{\;\mathrm{hv}\;}{\to }\;2\cdot \;\mathrm{OH}$$

(7) $$h^{+}+OH^{-}\;\overset{\;}{\to }\;\cdot \;\mathrm{OH}$$

In which, the ·OH and $\;h^{+}$ are strong oxidant which could degrade the NO_x_,

(8) $$\mathrm{NO}+\cdot \;\mathrm{OH}+\;\overset{\;}{\to }{\;\mathrm{HNO}}_{2}$$

(9) $${\mathrm{HNO}}_{2}+\cdot \;\mathrm{OH}\;\overset{\;}{\to }\;NO_{2}+H_{2}O$$

(10) $$NO_{2}+\cdot \;\mathrm{OH}\;\overset{\;}{\to }\;HNO_{3}$$

(11) $${\mathrm{NO}+O}_{2}^{-}\;\overset{\;}{\to }{\;\mathrm{NO}}_{3}^{-}$$

### 2.3. Experimental Equipment

#### 2.3.1. Photocatalytic Reaction Chamber

The NO and NO_2_ gases used in this experiment were produced by Changzhou Jinghua Industrial Gas Company (Changzhou, China) with a purity of 99.9%. In this research, the Taiwan Luchang UVA365 ultraviolet (UV) irradiator (Taiwan, China) was used to measure UV radiation, the measurement wavelength is 365 nm, the measurement range is 0–199 W/m^2^, and the resolution is 1 W/m^2^.

In this research, the photocatalytic environmental chamber was developed. The system contained a UV radiation system with variable intensities and a gas detection system that is able to accurately control and determine the gas during the whole photocatalytic reaction process [36,37].

- Structure of the test system

**Figure 2:**
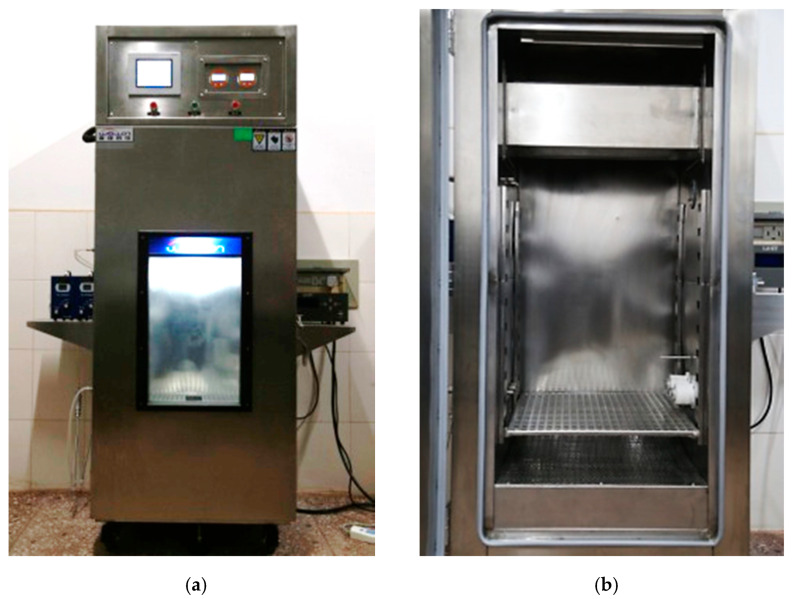
Overall structure of photocatalytic reaction test system; (**a**) external structure; (**b**) internal structure.

The external dimension of the environmental chamber is 70 × 70 × 170 cm^3^ as shown in Figure 2a. The inner size of the environmental chamber is 50 × 50 × 115 cm^3^, and the size of the UV light source slot is 50 × 18 × 16 cm^3^ as shown in Figure 2b.

The environmental chamber has good air-tightness performance, the light source is UV-LED light tubes with ARM controller processor, which is able to provide constant current and temperature monitoring. The radiation intensity of the light source can be adjusted in the range of 0–100%. In order to evaluate the effect of the reversible reaction of NO_2_ and N_2_O_4_ on the catalytic degradation process, and N_2_O_4_ gas analyzer was also installed in the environmental chamber.

#### 2.3.2. Light Intensity

In the design of the NO_x_ degradation test, the light intensity and distribution inside the photocatalytic reaction chamber should be close to the UV environment in the actual condition. The UV in the sunlight is mainly the UVA long wave band, so the LED light source selected in this test system emits UVA in order to simulate the actual sunlight condition.

**Figure 3:**
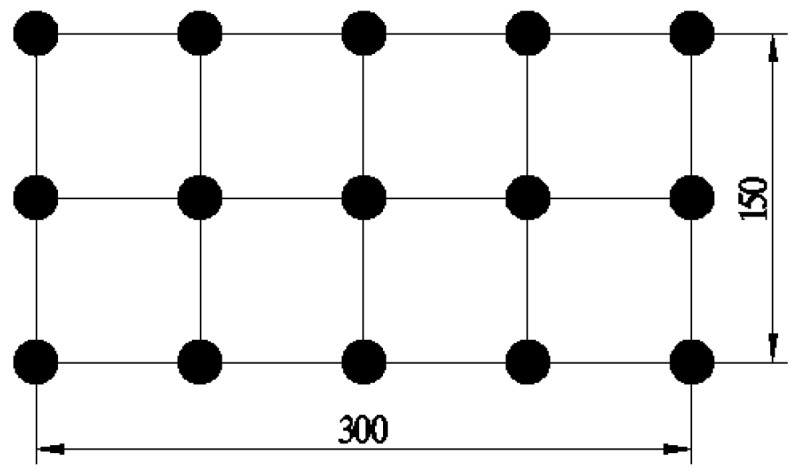
Layout of measuring points (unit: mm).

The irradiation intensity verification was also carried out for the light source, the test range was horizontally at the bottom of the chamber at the size of 300 × 150 mm^2^. The layout of the verification points are as shown in Figure 3.

When testing the degradation performance of asphalt pavement coating materials, the distance between the light source and the test specimen was 65 cm, and when testing the degradation performance of the roadside coating, the distance between the light source and the sandwich plywood was 70 cm. The irradiance intensity values at different irradiance distances of 65 cm and 70 cm were measured and it was found that this light source can effectively simulate the irradiance intensity of different levels of UV rays, and can be used to evaluate the intensity of UV in different regions or in different seasons.

#### 2.3.3. Gas Concentration

In order to determine the initial concentration of NO_x_ in the test process, three typical road sections near the bus station or the traffic light intersection were selected on the main road of Guangzhou (China) for monitoring, which including the road surface under the overpass, the road in the tunnel, and the uphill section. The reason to choose those three sections is that the car usually in the low-speed or idle state, and the incomplete combustion of gasoline leads to the high concentration of exhaust pollutants.

- Point M-1

**Figure 4:**
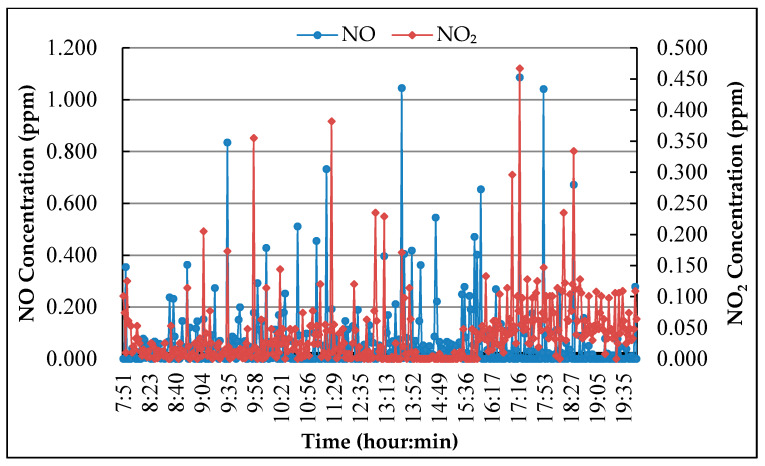
NO and NO_2_ concentration monitoring result.

The measuring point M-1 is located at the bottom of the overpass. The main road is a two-way eight-lane road with large traffic flow. There is a traffic light intersection about 100 m southwest of the measuring point, and a bus station about 20 m northeast of the measuring point. The monitoring result of NO and NO_2_ concentration in the air during the monitoring period is shown in Figure 4.

- Point M-2

**Figure 5:**
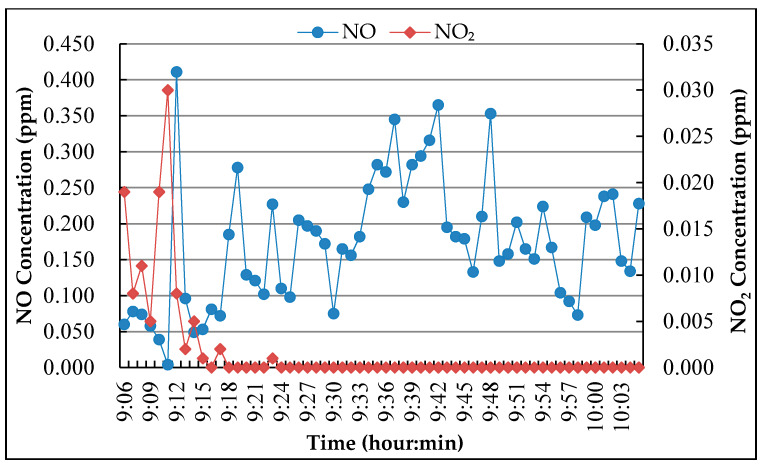
NO and NO_2_ concentration monitoring result.

The measurement point M-2 is located about 100 m inbound close to the end of a long tunnel with heavy traffic. The concentrations of NO and NO_2_ pollutants during the monitoring period are shown in Figure 5.

- Point M-3

**Figure 6:**
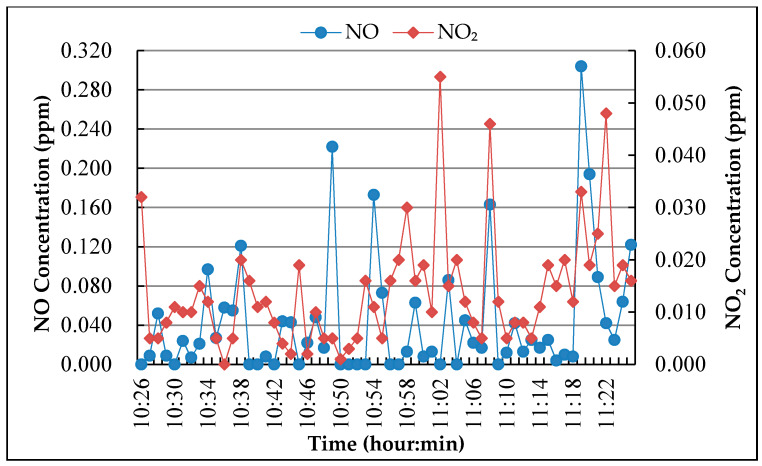
NO and NO_2_ concentration monitoring result.

The measurement point M-3 is an open location on an uphill road with medium traffic flow. The concentrations of NO and NO_2_ pollutants during the monitoring period are shown in Figure 6.

According to the field observation, the locations M-1 and M-3 have serious engine idling because there are a lot of buses parked near measuring point M-1, and measuring point M-3 is the uphill road section. While the measuring point M-2 has poor air circulation because it is located in the tunnel. As motor vehicle exhaust is one of the main sources of NO, the NO concentration peak was caused by the exhaust emissions because of the deceleration or idling of vehicles. After a period of time, NO emissions oxidize into NO_2_ which caused the peak of NO_2_. In the tunnel, due to the low oxygen concentration and the small amount of NO oxidized, the concentration of NO_2_ is not high, and it has been kept at low value for a long time.

In the degradation test of pavement surface coating, the concentration of NO was evaluated because the vehicle exhaust is near the ground, and in the degradation test of roadside coating, NO_2_ concentration was chosen as the evaluation index because NO was oxidized into NO_2_ when it was discharged into the air. According to the monitoring results, the initial concentration of NO and NO_2_ for the experiment was chosen as 0.500 ± 0.010 ppm.

### 2.4. Experimental Steps

The steps for the photocatalytic degradation test described are as follows: First, we put the asphalt mixture test specimen coated with nano-TiO_2_ surface coating material or the sandwich plywood test piece coated with the roadside coating material in the environmental chamber. Then, we turned on the temperature programmable controller and set the reaction temperature of 27 ℃, the temperature was monitored through the whole process. After the reaction temperature was stable for 4 h, the desired amount of NO_x_ was injected into the environmental chamber. Next, we recorded the NO_x_ concentration every 5 min. After the gas concentration value is stable, we turned on the UV light and set the irradiation intensity percentage, and then continued to record the change of NO_x_ concentration. 

In order to evaluate the impact of coating material on the skid resistance of asphalt pavement, the impact of coating amount on the texture depth (TD) evaluated, the coating amount was evaluated in the range of 0 g/m^2^ to 400 g/m^2^, the TD was measured following the Specifications for Design of Highway Asphalt Pavement: JTGD50-2006 [38]. The TD of pavement surface is an important index of pavement roughness, which refers to the average depth of uneven opening pores on a certain area of road surface, and it is mainly used to evaluate the macro roughness, drainage performance and skid resistance of pavement surface.

## 3. Results and Discussions

### 3.1. Experimental Analysis of the Degradation of Road Surface Coating

#### 3.1.1. Effect of Different Silane Coupling Agents on NO Degradation

**Figure 7:**
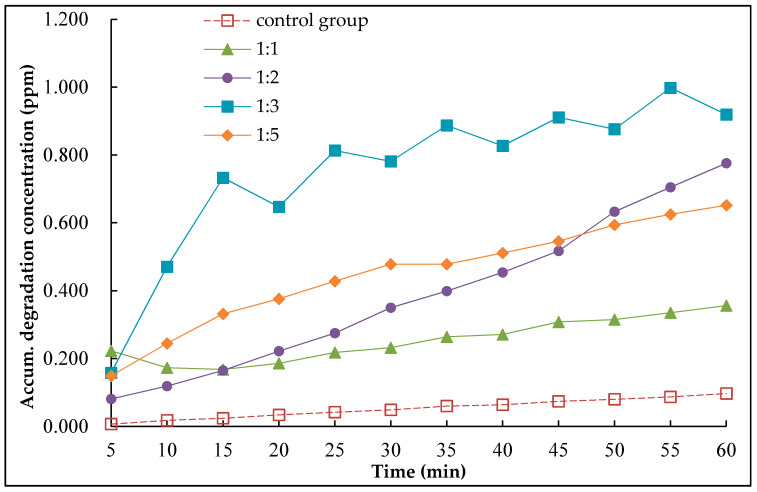
Test results of degradation with different amount of silane coupling agent.

In order to evaluate the impact of different silane coupling agents on the NO degradation performance, four samples with mass ratio of nano-TiO_2_ and silane coupling agent of 1:1, 1:2, 1:3 and 1:5 were compared with control samples. The test result is shown in Figure 7, where the X-axle was the time and the Y-axle was the accumulated degradation concentration. 

There is no nano-TiO_2_ surface coating material for the control group. It can be seen from Figure 7 that the NO is still reduced to a very small amount for the control group, which indicates that there is a certain natural degradation for the NO by itself. The NO degradation effect is obvious when the nano-TiO_2_ surface coating material was used, with the result indicating that the material can effectively degrade the NO in the road environment.

When the mass ratio of nano-TiO_2_ to silane coupling agent increased from 1:1 to 1:2, and from 1:2 to 1:3, the degradation effect of NO on the surface coating material is significantly enhanced, which shows that the increase of silane coupling agent can improve the degradation performance of the material.

When the mass ratio of nano-TiO_2_ and silane coupling agent increases from 1:3 to 1:5, the degradation effect of NO on the surface coating material is decreased, which indicate that there is an optimum amount of silane coupling agent. The reason is that when the silane coupling agent is too much, it greatly improved the adhesion between of nano-TiO_2_ and asphalt mixture, and thus reduced the reaction area between nano-TiO_2_ and NO, thus leading to the photocatalytic activity of nano-TiO_2_ decreased. Therefore, during practical application, it is suggested that the optimum mass ratio of nano-TiO_2_ to silane coupling agent is between 1:2 and 1:3.

#### 3.1.2. Photocatalytic Recovery of Nano-TiO_2_ Road Surface Coating Materials

**Figure 8:**
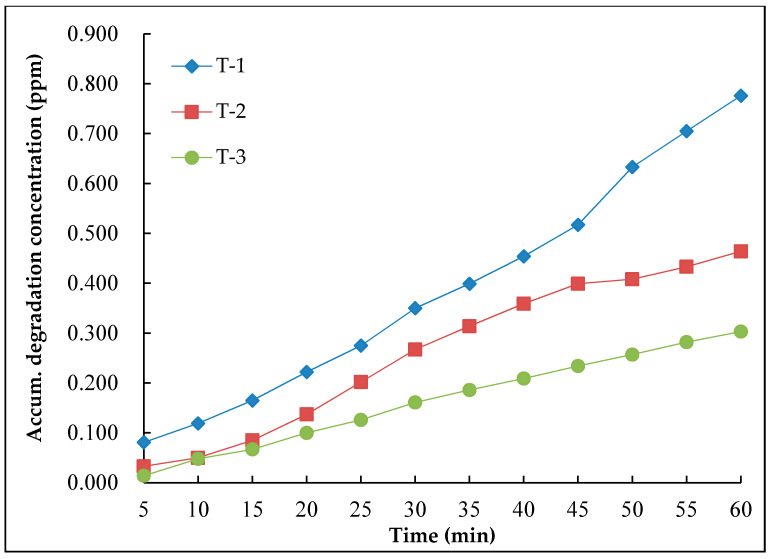
Consecutive degradation results of the same specimen.

In order to evaluate the photocatalytic recovery properties of nano-TiO_2_ road surface coating materials, the sample with the dosage ratio of nano-TiO_2_ and silane coupling agent of 1:2 was selected to conduct the recovery test. The same sample coated with the material was tested for three times consequently, and the test results are shown in Figure 8. In Figure 8, the Y-axle was the accumulated degradation concentration, T-1 means the result after the first record, T-2 means the result after two tests and T-3 means the result after three tests.

It could be seen from Figure 8 that after continuous degradation test on the same test piece, the degradation effect of the coating material on NO was gradually weakened. The reason was that the mineralized salt and inorganic salt was generated during the photocatalytic reaction, and which covered the surface of the coating material to decrease its photocatalytic activity. With the increase of the coating thickness, the photocatalytic performance of nano-TiO_2_ was affected.

**Figure 9:**
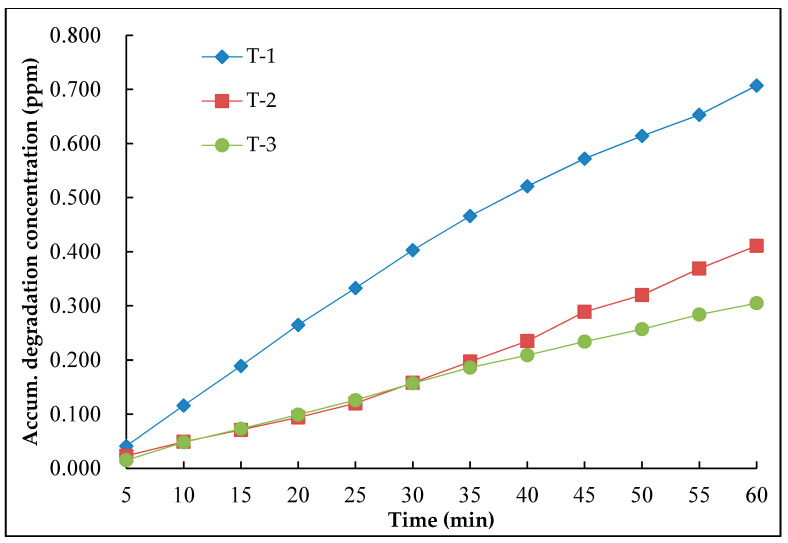
Degradation test results after cleaning.

For this reason, the deionized water was used to clean the surface of the test piece after the test, and then carry out photocatalytic degradation test after drying. The test results were shown in Figure 9, where Y-axle was the accumulated degradation concentration. It can be seen from Figure 9 that when deionized water was used to clean and dry the sample after three consecutive degradation tests, the photocatalytic activity was restored to a great extent. Then with the increase of the number of tests, the photocatalytic degradation effect is gradually reduced, which indicated that the photocatalytic activity of the coating material can be restored, and is suitable for field applications.

#### 3.1.3. Effect of Different Irradiation Intensity on Photocatalytic Degradation Efficiency

**Figure 10:**
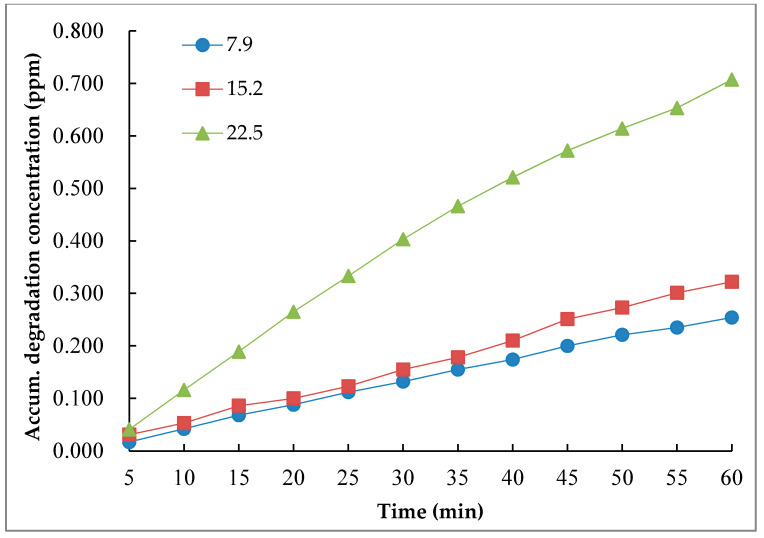
NO degradation test results under different irradiation intensity.

As the UV radiation intensity is different at different regions or in different seasons, it is necessary to evaluate the influence of different radiation intensity on the degradation performance of nano-TiO_2_ road surface coating materials. The dosage ratio of nano-TiO_2_ and silane coupling agent of 1:2 was used for the evaluation. In order to simulate different UV intensity in field, adjust the light source intensity ratio to 5%, 9% and 13% when the UV radiation distance was 65 cm, which is corresponding to the UV radiation intensity of 7.9, 15.2 and 22.5 W/m^2^ respectively. Those three light intensities can simulate the level 2, 3 and 4 UV radiation intensities in actual conditions. The test result of different intensities on the photocatalytic degradation efficiency is shown in in Figure 10, where Y-axle was the accumulated degradation concentration.

It can be seen from Figure 10 that the degradation performance of nano-TiO_2_ coating material was enhanced with the increase of irradiation intensity of light source. When the irradiation intensity increased from 7.9 W/m^2^ to 15.2 W/m^2^, the degradation effect of the gas was not significantly improved. When the irradiation intensity increased from 15.2 W/m^2^ to 22.5 W/m^2^, the degradation effect of the NO was significantly enhanced. The UV intensity of 22.5 W/m^2^ is corresponding to the UV level 4, which is mostly occurred in summer or in plateau area. Therefore, the selection of different dosage according to the actual situation of the local UV intensity can achieve better applicable performance.

#### 3.1.4. Influence of Surface Coating Materials on Skid Resistance of Pavement

**Figure 11:**
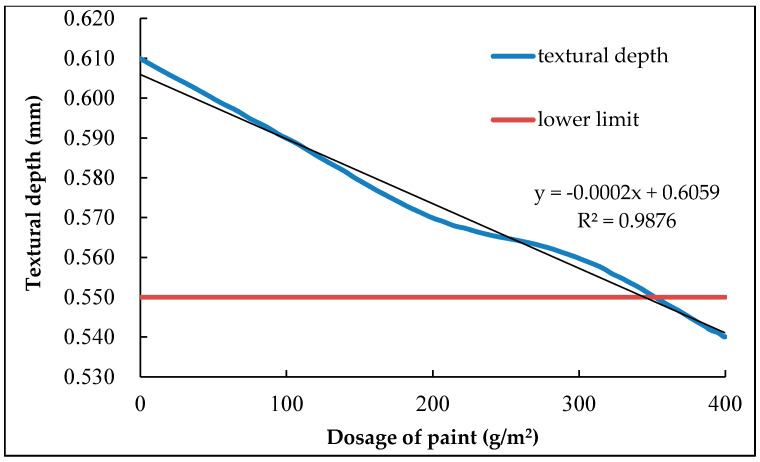
Impact of coating amount on the pavement texture depth (TD).

The skid resistance of pavement needs to be evaluated as the coating material going to be painted on the surface layer of asphalt pavement, the textural depth (TD) was used to evaluate the influence of the coating amount on the skid resistance performance. The result of textural depth is shown in Figure 11.

It can be seen from Figure 11 that the TD of the asphalt mixture surface was reduced with the increase amount of coating, which indicating that the coating material have an adverse effect on the skid resistance of the pavement. In order to ensure that the coating material did not affect the driving safety of the road, the minimum standard requirement of the TD was determined as 0.55 mm according to the engineering experience. Therefore, the maximum limit of the coating amount is suggested to 350 g/m^2^. When the coating amount was lower than the amount, it is considered that the sliding resistant performance of asphalt pavement still meets the driving requirements.

### 3.2. Degradation Evaluation of Roadside Coating

#### Effects of Different Surfactant Dosages on NO_2_ Degradation

**Figure 12:**
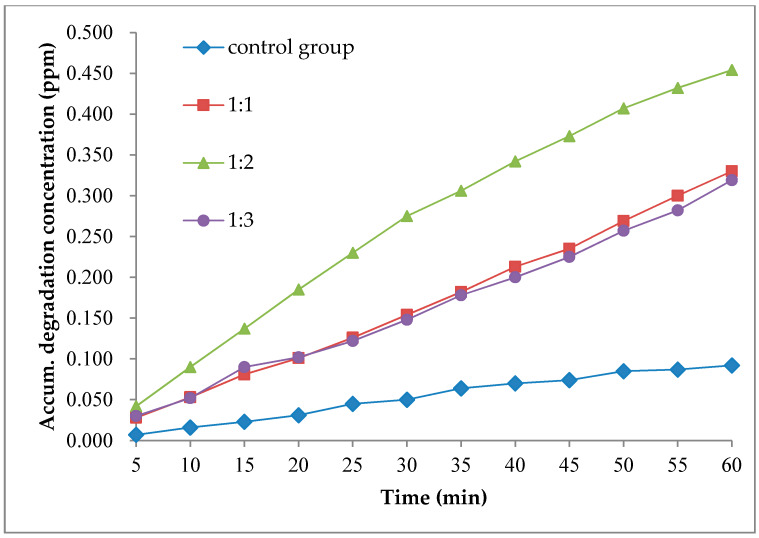
Degradation test results of NO_2_ with different surfactant dosage.

In this research, the sodium dodecylbenzene sulfonate was used as surfactant to reduce the agglomeration problem of nanoparticles and improve the interfacial adhesion between the coating material and roadside plates. Three different surfactant contents, of 1:1, 1:2, and 1:3 were evaluated and compared with the control group without surfactant added. The rest result is shown in Figure 12, where the Y-axle was the accumulated degradation concentration. 

Through the comparison of three tests with different content of surfactant and the control group, it can be seen that when the nano-TiO_2_ roadside coating material is not used, the content of NO_2_ still decreased to a small certain extent which indicated that the NO_2_ undergo a certain degree of natural degradation by itself. When the nano-TiO_2_ roadside coating material was used, the degradation effect of NO_2_ was obviously improved, which shows that the coating material can effectively achieve the degradation of NO_2_ near the road.

The result also has shown when the mass ratio of nano-TiO_2_ to surfactant increased from 1:1 to 1:2, the degradation effect of roadside coating materials on NO_2_ was significantly enhanced, which shows that the increase of surfactant was helpful to improve the degradation performance of roadside coating materials. And when the mass ratio of nano-TiO_2_ to surfactant increased from 1:2 to 1:3, the degradation effect of roadside coating materials on NO2 is not enhanced but decreased, which indicated that there is an optimum amount of surfactant additive. The reason was that although the surfactant helps to solve the agglomeration problem of nano-TiO_2_, the surface activation energy of nano-TiO_2_ particles was also reduced which lead to the degradation of photocatalytic performance. Overall, based on the analysis, the recommended mass ratio of nano-TiO_2_ to sodium dodecylbenzene sulfonate was about 1:2.

## 4. Conclusions

A photocatalytic test system with controllable environmental parameters that can simulate the whole process of the photocatalytic reaction was developed in this research. The photocatalytic degradation effect of nano-TiO_2_ road surface and roadside coating materials under different amounts of silane coupling agent and different UV irradiation intensities were evaluated based on the field measured automobile exhaust gas concentration. Based on the analysis, the following conclusions can be drawn:

1. Both the pavement surface and the roadside coating material can effectively degrade the NO_x_ in the road environment.
2. It suggested that the optimum mass ratio of nano-TiO_2_ to silane coupling agent was between 1:2 and 1:3, and the optimum mass ratio of nano-TiO_2_ to sodium dodecylbenzene sulfonate was about 1:2 in the field application. Additionally, the maximum coating amount of 350 g/m^2^ is suggested in order to ensure the skid resistance of the pavement surface.
3. The results have shown the degradation effect of coating materials on NO was gradually weakened, and that with the increasing number of experiments, and catalytic activity of the coating material can be restored after washed by deionized water.
4. With the increase of UV irradiation intensity, the degradation performance of nano-TiO_2_ coating material was enhanced. Therefore, when the coating material was used in different areas, it is necessary to determine the optimum dosage according to the actual situation of the local UV intensity.
5. The skid resistance of asphalt pavement is reduced with the increase of coating amount, in order to ensure enough skid resistance performance of asphalt mixture, it is suggested that the maximum coating amount is 350 g/m^2^.

## Figures and Tables

**Table 1:** Technical indexes of VK-TA18 nano-TiO_2._

Properties	Results
Appearance	White power
Hydrophilic coefficient	0.56
Specific surface area (m^2^/g)	60–120
Drying weightlessness 105℃,2 h	≤1.0%
Ignition loss	≤1.0%
Purity	≥99.8%
pH value	8.1
Crystal	Anatase
